# Morphology, molecular phylogeny and record verification of *Sarcophaga ruficornis* Fabricius (Diptera: Sarcophagidae) from Egypt

**DOI:** 10.1038/s41598-025-25934-0

**Published:** 2025-11-18

**Authors:** Gawhara M. M. Abu El-Hassan, Reham H. Abo El-Ela, Rabab Sawaby, Hayam El-Hamouly, Bahira M. El Sawaf, Enas H. Ghallab

**Affiliations:** Department of Entomology, Faculty of Science, Ain Shams University, Abbassia, Cairo, 11566 Egypt

**Keywords:** DNA barcoding, Phylogeny, Flesh flies, Taxonomy, COI, Genitalia

## Abstract

In forensic entomology, accurate species identification is essential for calculating the exact minimum postmortem interval (minPMI), as insect developmental rates are highly species-specific. *Sarcophaga (Liopygia) ruficornis* (Fabricius, 1794) is a species of medical and forensic significance. Recently, it was recorded from Egypt for the first time. The current study aimed to conclusively identify *S. ruficornis* in Egypt through DNA barcoding and morphological examination of both adult males and females, for the first time, to our knowledge. A segment of the mitochondrial cytochrome oxidase subunit one gene (COI) of *S. ruficornis* was amplified, and a sequence length of 817 bp was obtained and submitted to GenBank. The genitalia of both sexes and the diagnostic morphological characters of the species were examined and illustrated. In addition, the phylogenetic relationship of the Egyptian population of *S. ruficornis* was investigated. This study demonstrates the utility of DNA barcoding for investigating the genetic composition and variation of *S. ruficornis* populations and provides essential data for the identification of *S. ruficornis* in Egypt, which makes it possible to identify a specimen correctly even when only limited morphological evidence is available.

## Introduction

Sarcophagidae have a significant role in the decomposition of corpses because several of their species are among the first to arrive at a corpse^1^. The genus *Sarcophaga* Meigen, 1826 (flesh flies) belongs to the family Sarcophagidae under the order Diptera and includes 890 recognized species classified into 169 subgenera. It is widely distributed, particularly in the Old World^2,3^.

One of the most significant necrophagous flesh flies attracted to corpses at any level of decomposition, from fresh to advanced, is *Sarcophaga ruficornis*, which was recently recorded in Egypt^4^. It has been documented on decomposing fish, chicken, and rabbit carrion. It breeds in cadavers and excrement. It is distributed worldwide^5^. As far as we know, there is no information available regarding the genetic variations among the populations of this species in Egypt.

Although morphological identification methods are considered the most convincing traditional way to identify insects, morphological similarity in certain species poses identification challenges as they require considerable time, holotype comparison, and taxonomic expertise^6,7^. Nevertheless, because so many species in this genus share some anatomical characteristics, it might be difficult to identify *Sarcophaga* at the species level, particularly for the females^8,9^. Typically, the genitalia of males are used for the morphological identification of the species. However, the improper preservation of specimens obtained from forensic investigations frequently makes it challenging to observe these features^10^.

On the other hand, molecular identification methods are rapid and ultra-sensitive. They can be performed on mounted specimens and on any stage of the life cycle without additional rearing. Also, DNA identification is fairly unaffected by preservation methods^11^.

One of the most promising molecular methods is DNA barcoding, a molecular tool widely used to detect interspecific and intraspecific variation in DNA sequences of specific genes, providing a powerful approach for species identification^7,12^. In most instances, mitochondrial gene sequences, specifically a short, standardized region of the genome, are utilized^13,14^. The COI is one of the primary markers employed to distinguish among cryptic, morphologically similar, and closely related animal species^7,10,13,15^. The COI barcodes have proven to be an efficient alternative means for the identification of species of flesh flies^1,8,10,14–22^.

In fact, the use of molecular DNA-based identification for some species not only complements traditional morphological identification but can also provide insights into population structure^18,23^. Accordingly, the current study aims to analyze the first mitochondrial COI sequences (DNA barcodes) of *S. ruficornis* from Egypt, identify the genetic variation within its Egyptian populations, and report as well as illustrate the species’ morphological characteristics.

## Methods

### Fly collection and rearing conditions

Specimens were collected from a garden in Abbassyia, Cairo, Egypt. Flesh fly females were captured individually in Falcon vials in July 2022. A piece of rotten buffalo meat was used as bait and placed in an open area under direct sunlight. All sampled females were reared separately until oviposition, thereby producing pure colonies of the same species. After the emergence of adults, males of *S. ruficornis* were examined and identified at the species level based on the following external morphological characters: antennae with the 2nd and 3rd segments at least partly yellow, gena with yellowish white setae, and terminalia reddish color, as well as the structure of male genitalia. Then, a perpetual colony of *S. ruficornis* was maintained in an insectary. The colony was reared on fresh buffalo meat and a saturated sucrose solution under controlled conditions, including a 12:12 LD photoperiod, 28 °C, and 65% relative humidity.

### Morphological examination:

Flies were killed using a killing jar containing ethyl acetate and then pinned through the right side of the mesothorax using standard entomological pins. To examine the male genitalia, the abdomen was gently detached using fine forceps and pins. It was then immersed in 10% KOH overnight to soften the chitin and remove any unwanted parts, and then washed in distilled water. The genitalia were carefully dissected from the abdomen, examined in detail, and identified based on the characters described by Suwannayod et al., Sharma et al., Buenaventura and Pape, and Ramos et al.^3,5,24,25^. The female genitalia were examined using the same technique. The terminology for characteristic features of female genitalia followed that of Zhang et al.^26^. The genitalia of both sexes were photographed from different angles using a Tobo USB 2.0 digital microscope. Other diagnostic morphological characters, such as the head, antennae, and wings, were examined and photographed.

### Molecular identification

#### Isolation, amplification, and sequencing of the COI marker gene

Molecular identification of the morphologically identified *Sarcophaga* flies was performed by targeting the COI gene as described by Abu El-Hassan et al.^4^. Genomic DNA was extracted from freshly anesthetized individual *Sarcophaga* flies stored at − 20 °C using the Qiagen DNeasy Blood & Tissue Kit (Cat. No. 69504, Germany), following the manufacturer’s instructions. The mitochondrial cytochrome c oxidase subunit I (COI) gene was targeted for amplification using the universal primers LCO1490 (5′-GGTCAACAAATCATAAAGATATTGG-3′) and HCO2198 (5′-TAAACTTCAGGGTGACCAAAAAATCA-3′), as described by Abu El-Hassan et al.^4^. PCR was performed in a final volume of 20 µL containing 1 × PCR buffer, 3.2 pmol of each primer, 200 µM dNTPs, 0.5 U HotStar Taq DNA Polymerase (Qiagen), and template DNA. Amplification was performed under the following thermal cycling profile: an initial denaturation at 94 °C for 30 s, followed by 35 cycles of denaturation at 94 °C for 30 s, annealing at 48 °C for 30 s, extension at 72 °C for 60 s, and a final extension at 72 °C for 10 min. To optimize the assay, a gradient PCR was conducted with annealing temperatures ranging from 55 to 65 °C for 40 cycles. Amplicons were resolved on 1.5% agarose gels stained with ethidium bromide (0.375 µg/mL), and HyperLadder II (Bioline) was used as a molecular weight marker.

The isolated sequence was compared with closely related sequences using BLAST (Basic Local Alignment Search Tool) via the NCBI (National Center for Biotechnology Information) platform (http://blast.ncbi.nlm.nih.gov/Blast.cgi), followed by multiple sequence alignment. Subsequently, species identification was performed based on the COI locus using BOLD (Barcode of Life Data Systems) analysis (http://www.boldsystems.org/). The amplified sequence was compared to the closely related sequences retrieved from the BLAST results and then subjected to multiple sequence alignment. Using the Clustal W Algorithm in MEGA 11 (version 11), COI sequences were aligned with a transition weight of 0.5, a delay divergence cutoff of 30%, a gap opening penalty of 15, and a gap extension penalty of 6.66^27^. The Maximum Likelihood (ML) approach was used to generate a phylogenetic tree from the multiple sequence alignments^28^. Pairwise genetic distances among sequences were calculated in MEGA 11 using the Kimura 2-parameter (K2P) model of nucleotide substitution with bootstrap testing (1000 replicates). *Chrysomya megacephala* (Family: Calliphoridae) was designated as the outgroup to root the tree, as it represents a taxon closely related to but clearly outside the Sarcophagidae family. The resulting pairwise distance values were used to assess the level of genetic differentiation among taxa and to evaluate whether the observed genetic distances are consistent with interspecific or intraspecific variation.

## Results

### Morphological identification

#### List of synonyms for *Sarcophaga ruficornis* (Fabricius, 1794)

- 1794. *Musca ruficornis* Fabricius, Ent. Syst., 4: 314.
- 1830. *Myophora fulvicornis* Robineau-Desvoidy, Mém. Pres. Div. Sav. Acad. R. Sci. Inst. Fr. 2:341.
- 1851. *Sarcophaga ruficornis*: Macquart, Mém. Soc. Sci. Agric. Arts, Lille, 206.
- 1928. *Liopygia friederichsiana* Enderlein, Arch. klassif phylogen. Ent., 1(1): 42.
- 1950. *Sarcophaga muspratti* Zumpt, Proc. R. Ent. Soc. London (B) 19:165.
- 1830. *Sarcophaga ruficornis*: Wiedemann, Aussereurop. zweifl. Jnsekt., 2: 361.
- 1945. *Parasarcophaga ruficornis*: Lopes, Revta bras. Bioi., 5(3): 401.
- 1963. *Parasarcophaga (Liopygia) ruficornis*: Rohdendorf, Stuttg. Beitr. Naturk., 124:1–22.

#### Diagnostic morphological characters

**Fig. 1:**
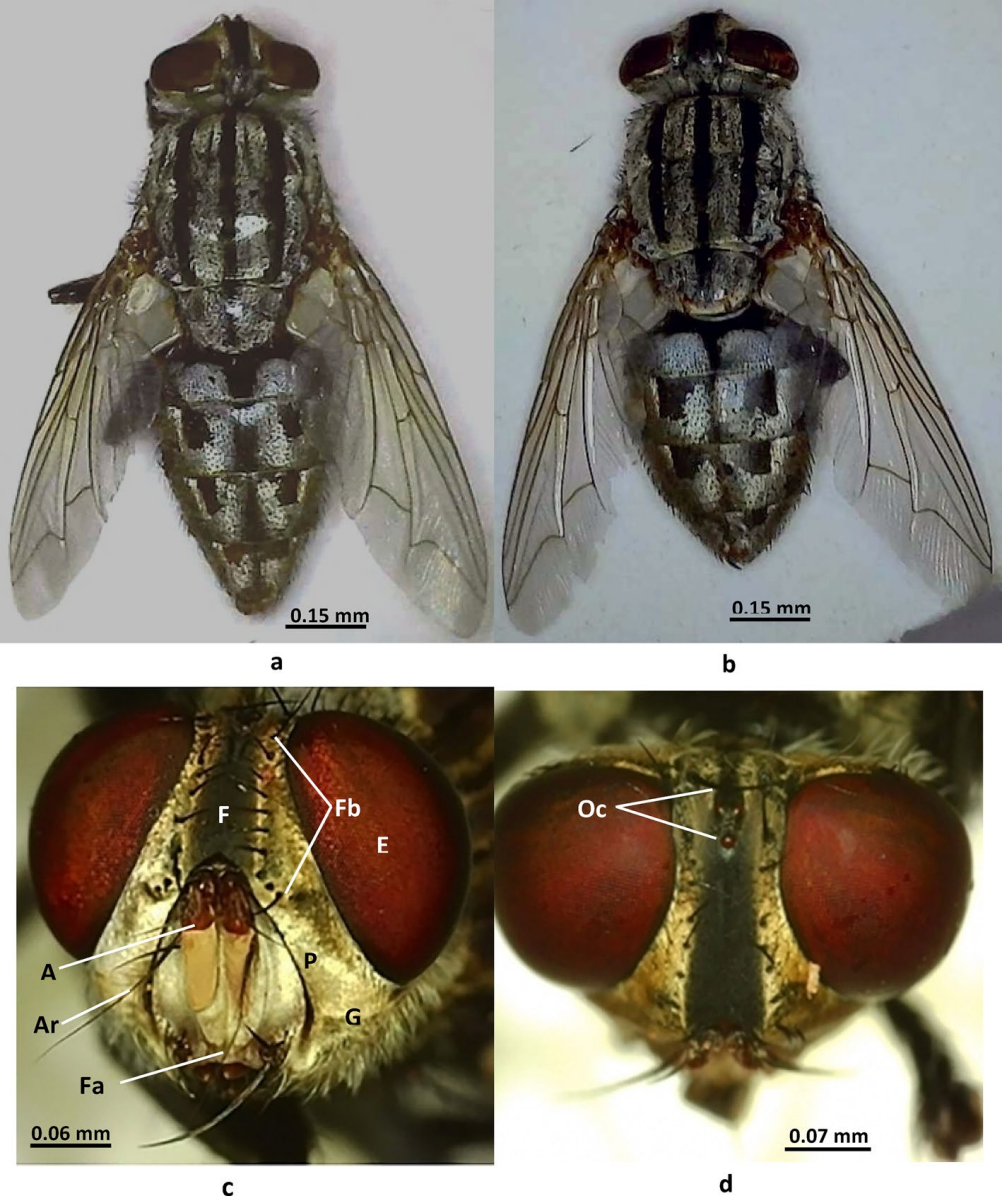
Adult morphology of *Sarcophaga ruficornis*. (**a**, **b**) Dorsal view: a male. b female. (**c**, **d**) Head details: c frontal view. d frontodorsal view. Abbreviations: A, antenna; Ar, arista; E, eye; F, frons; Fa, face; Fb, frontal bristles; G, gena; Oc, ocellar tubercle; P, parafacial.

**Body** length is 7–13 mm, with males slightly larger than females. The body is black and grey with a checkered abdomen (Fig. 1a, b).

**Fig. 2:**
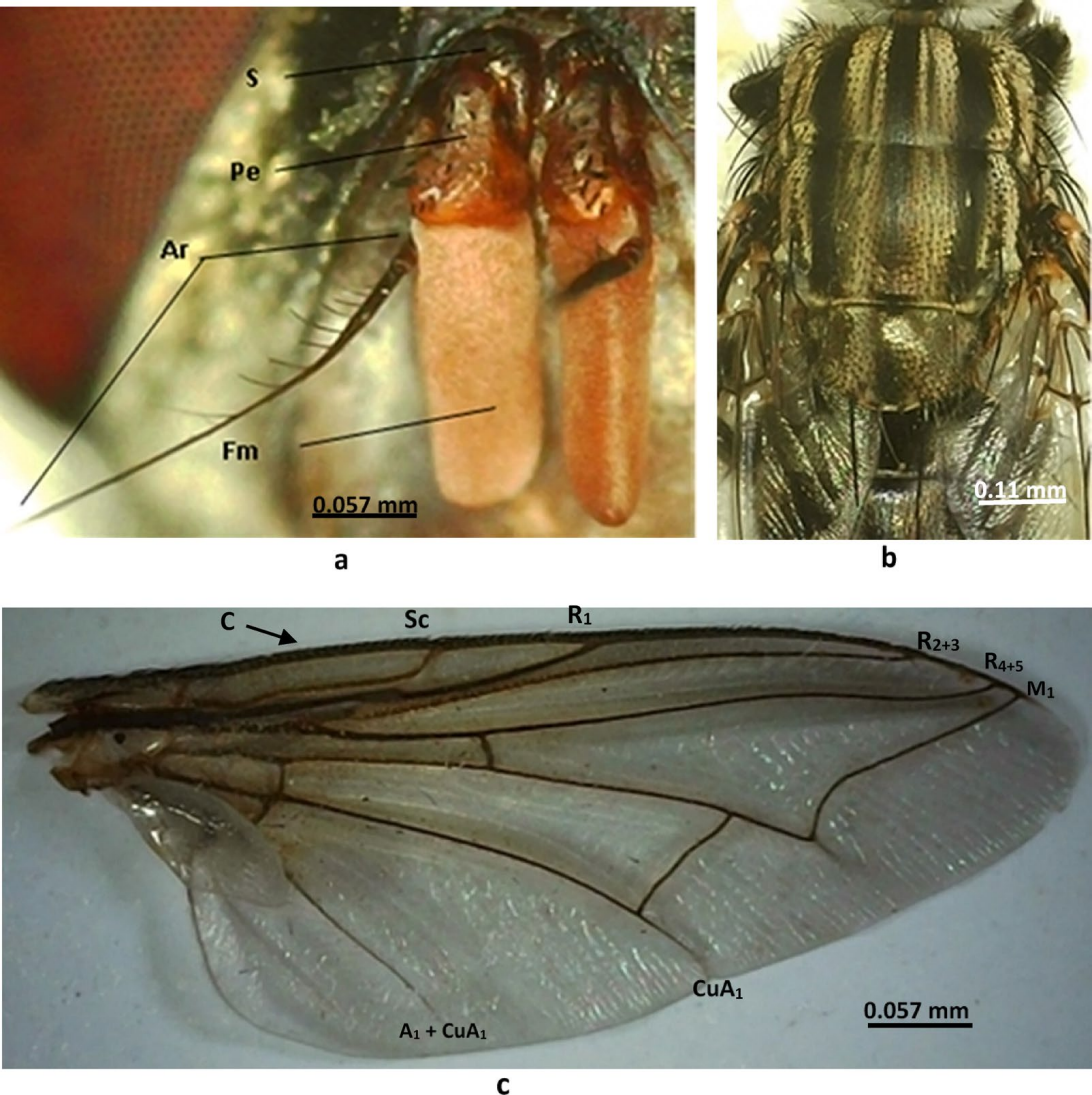
Morphological characters of *Sarcophaga ruficornis*. (**a**) Antenna. (**b**) Thorax, dorsal view. (**c**) Forewing. Abbreviations: A, anal vein; Ar, arista; C, costa; Cu, cubitus; F, flagellum; M, media; P, pedicel; R, radius; S, scape; Sc, subcosta.

**Head**: The frons width is about two-fifths the width of an eye; there are 12 frontal bristles; the facial ridge is light brown and bare; the parafacial is black with golden pollen; the gena is black, covered with silver pollen and yellow hairs; the post-gena is black covered with grey hairs (Fig. 1c); there is a black ocellar triangle with black hairs (Fig. 1d); the antennal flagellum is orange, the scape and pedicel are blackish brown, the arista is long and hairy on the basal half only (Fig. 2a); there is a short blackish proboscis.

**Thorax**: The thorax is black, covered with silver pollen, and has 3 black longitudinal stripes (Fig. 2b).

**Wings**: The wings are hyaline with brownish veins; the Radius vein 1 (R1) is bare; the costal spines are not stout (Fig. 2c).

**Fig. 3:**
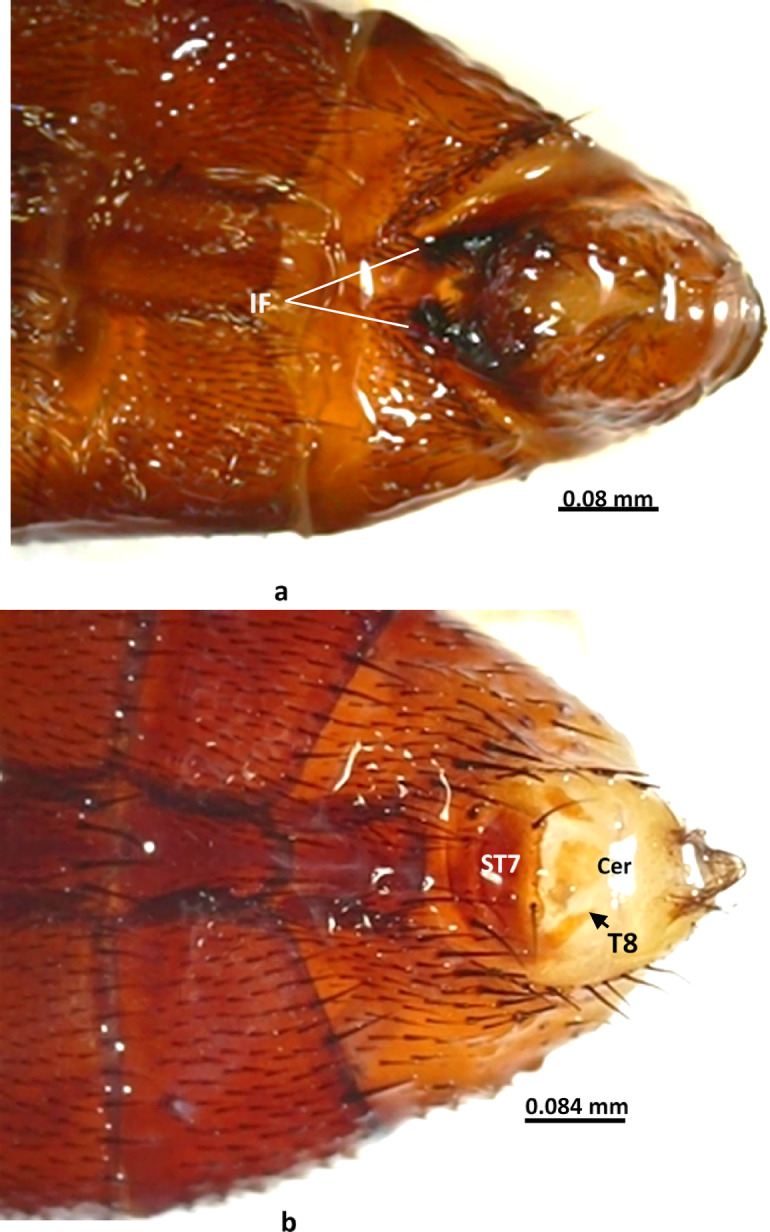
Ventral view of last abdominal ventrites of *Sarcophaga ruficornis*. (**a**) Male. (**b**) Female. Abbreviations: Cer, cercus; If, inner forceps; ST7, seventh sternite; T8, eighth tergite.

**Abdomen**: The abdomen is black with a silver checkered pattern, elongated with a truncated end in males (Fig. 1a) and broader with a tapered end in females when viewed dorsally (Fig. 1b). Males and females can also be differentiated, ventrally by the shape of the apical abdominal ventrites, where in the male the fifth ventrite is V-shaped, and the outer forceps are visible (Fig. 3a). In the female, the shape of the cerci and tergites is very characteristic, as shown in (Fig. 3b).

**Fig. 4:**
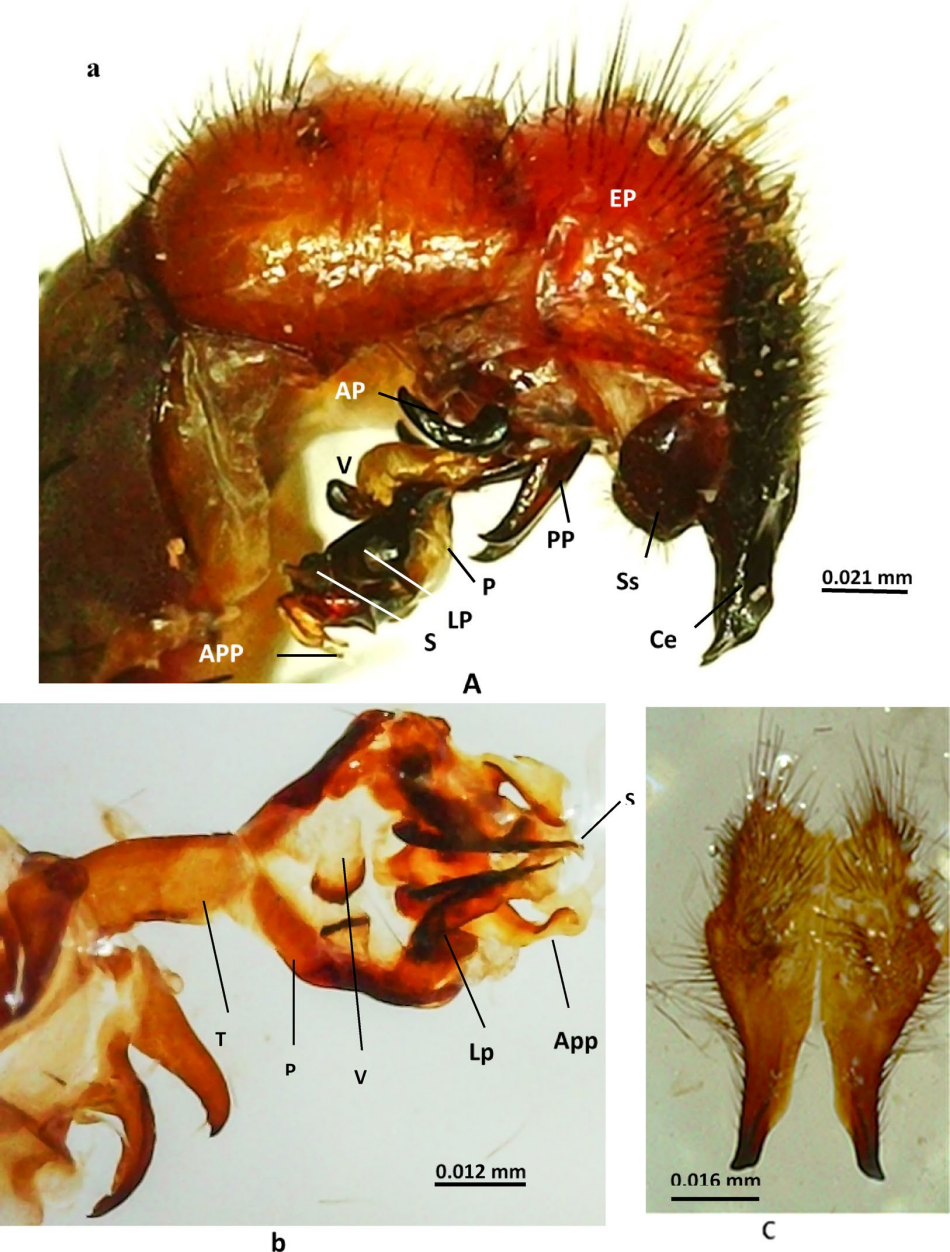
Male terminalia of *Sarcophaga ruficornis*. (**a**) Lateral view of male genitalia. (**b**) Ventral view of genitalia. (**c**) Cerci. Abbreviations: AP, anterior paramere; APP, apical plate of paraphallus; Ce, cercus; EP, epandrium; LP, lateral plate; P, paraphallus; PP, posterior paramere; S, stylus of glans; Ss, surstylus; T, theca; V, ventralia.

**Fig. 5:**
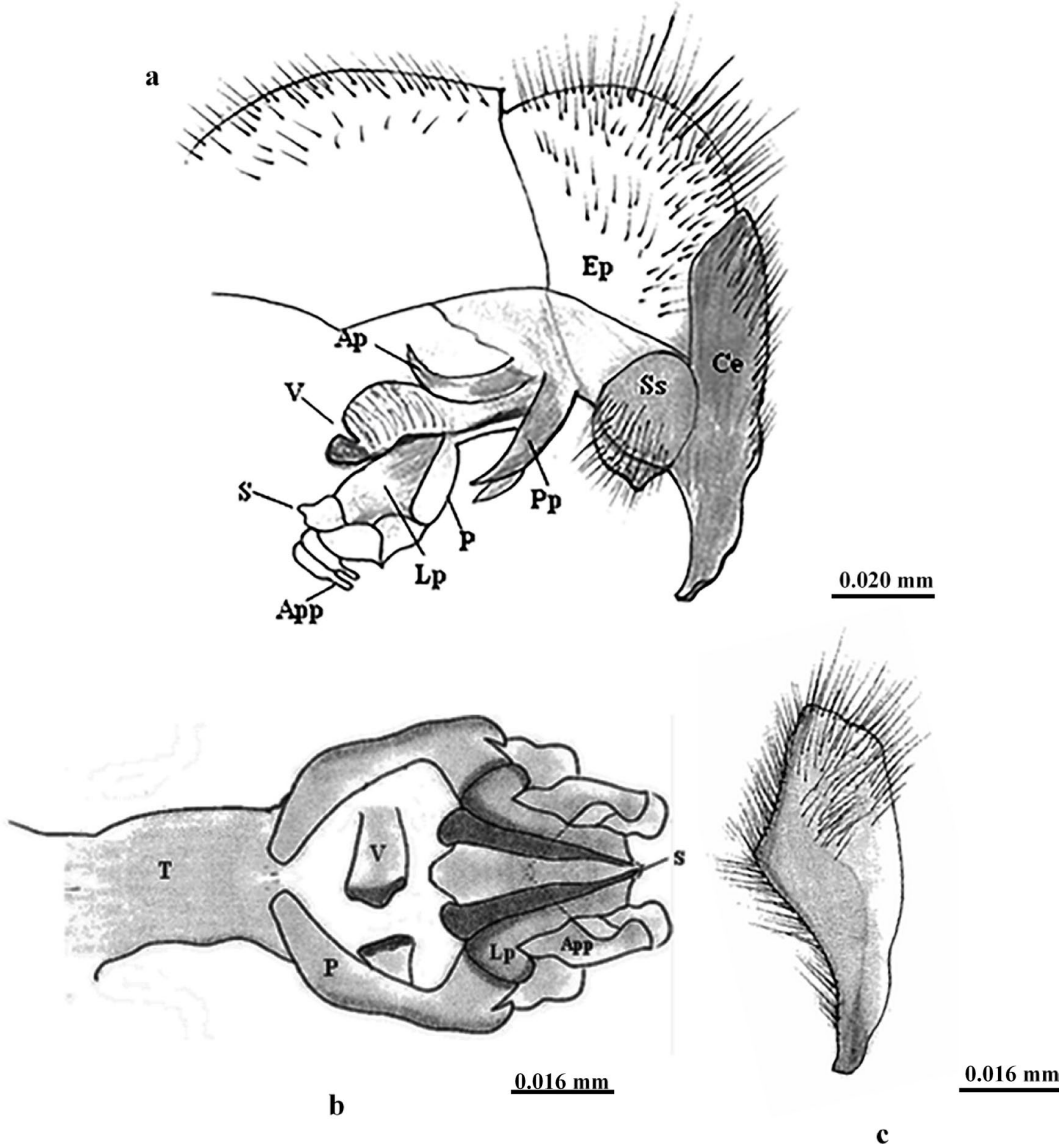
Diagrammatic illustration of the male terminalia of *Sarcophaga ruficornis*. (**a**) Lateral view. (**b**) Genitalia, ventral view. (**c**) Left cercus. Abbreviations: AP, anterior paramere; APP, apical plate of paraphallus; Ce, cercus; EP, epandrium; LP, lateral plate; P, paraphallus; PP, posterior paramere; S, stylus of glans; Ss, surstylus; T, theca; V, ventralia.

**Male terminalia** (Figs. 4 and 5): The first genital segment is reddish brown with long marginal bristles, the second segment has black hairs but lacks marginal bristles; the cerci are thickly covered with long hairs and end in an acute pointed tip; the surstylus is slightly oval with apical hairs; the anterior and posterior parameres are bent with pointed ends; the paraphallus is longer than the theca, and both are sclerotised; the apical plate of the paraphallus is not sclerotised and consists of two plates, one of which has backwardly prominent spines and a lateral plate that is small with a serrated anterior margin; the styli of glans consist of a pointed membranous part and another part that has a rosette-like structure; the ventralia are bulbous, more sclerotised at the end and have a long stalk and spinous sub-basal part.

**Fig. 6:**
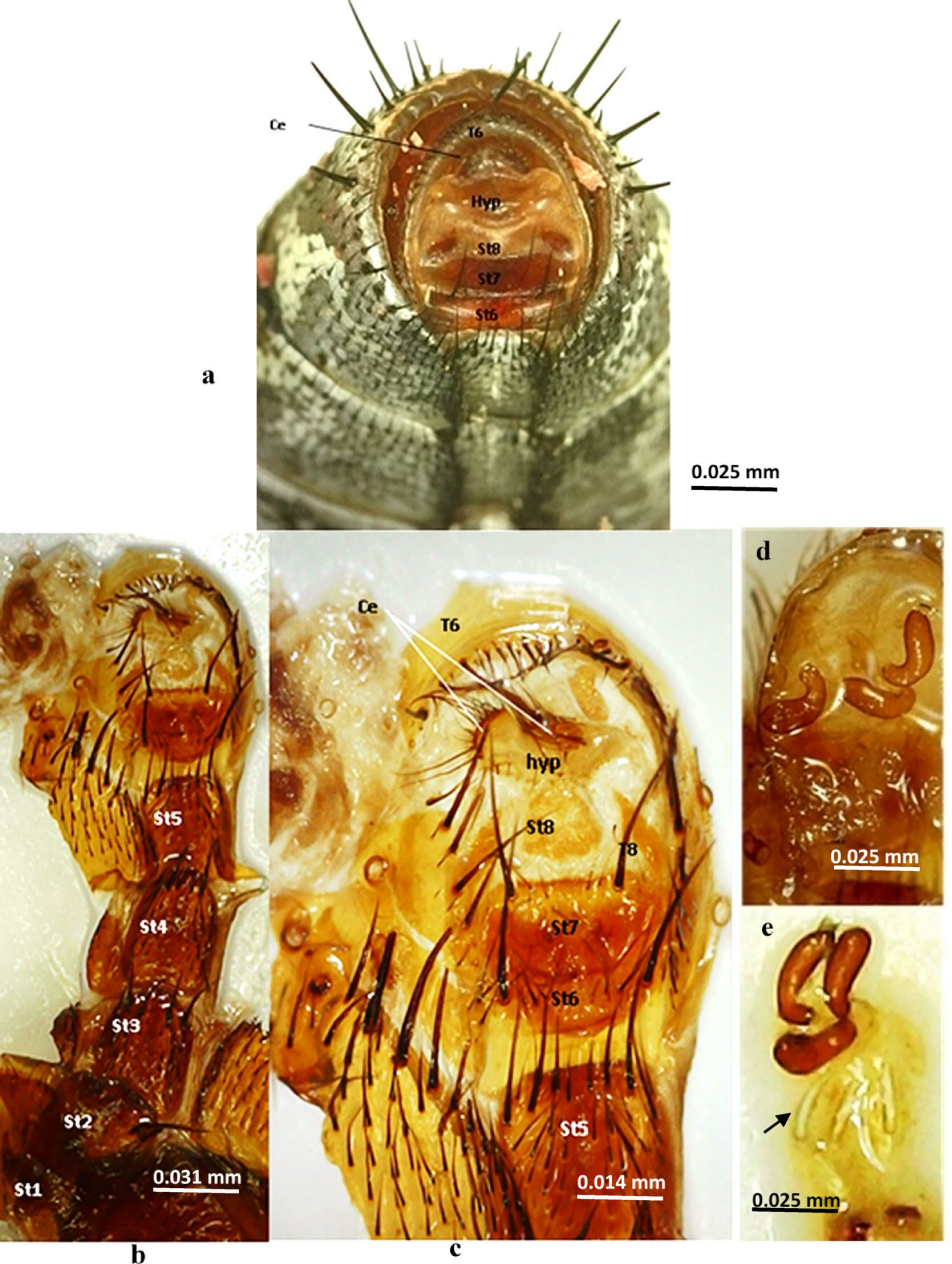
Female terminalia of *Sarcophaga ruficornis*. (**a**) Terminal abdominal segments, ventral view. (**b**, **c**) Caudal view. (**d**) Spermatheca. (**e**) Spermathecal ducts. Abbreviations: Ce, cercus; Hyp, hypandrium; T6, tergite 6; T8, tergite 8; St6, sternite 6; St7, sternite 7; St8, sternite 8.

**Fig. 7:**
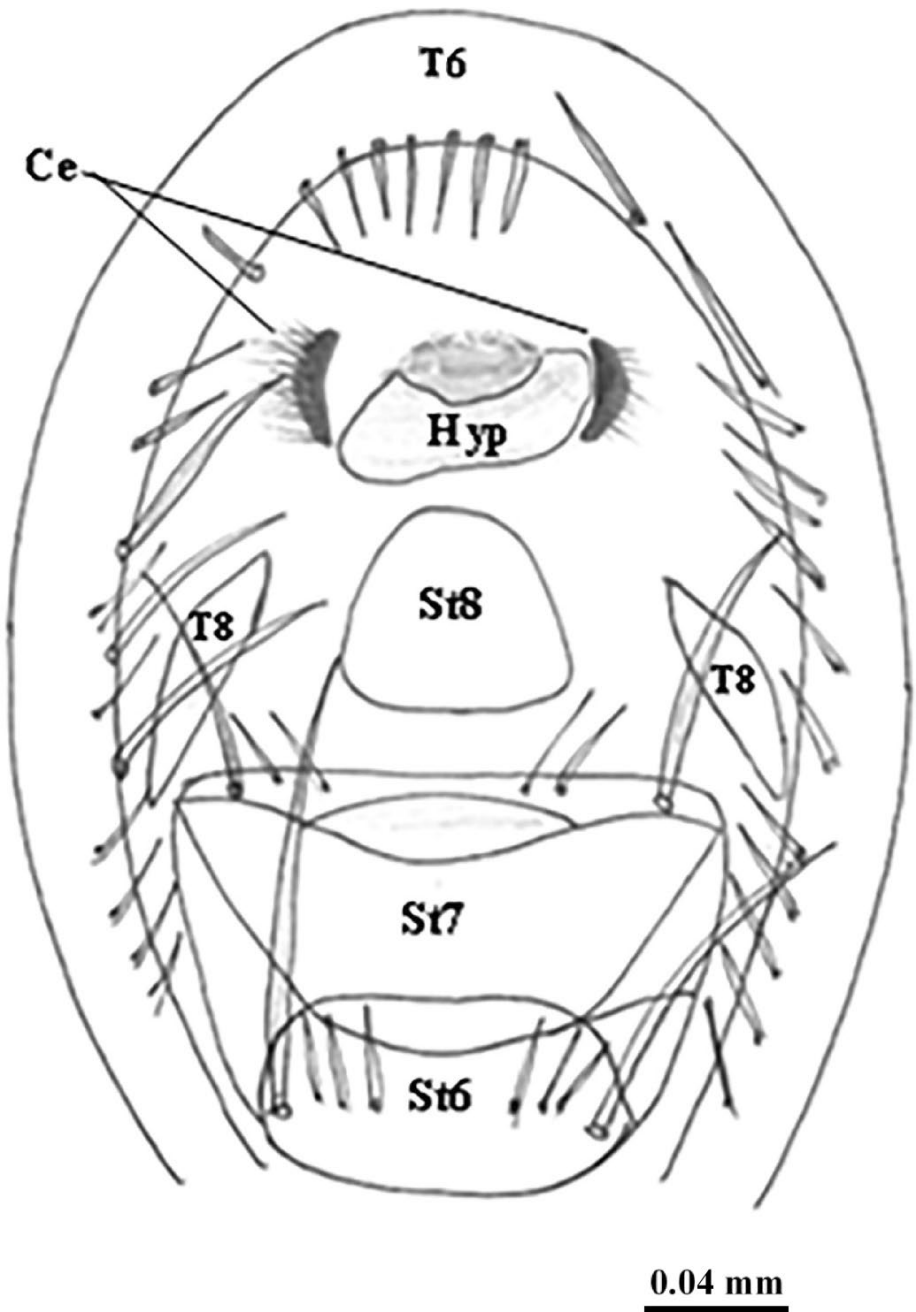
Diagrammatic illustration of caudal view of female terminalia of *Sarcophaga ruficornis*. Abbreviations: Ce, cercus; Hyp, hypandrium; T6, tergite 6; T8, tergite 8; St6, sternite 6; St7, sternite 7; St8, sternite 8.

**Female terminalia** (Figs. 6 and 7): Tergite 6 is undivided (Fig. 6a), the epiproct is not apparent, the hypoproct is not sclerotized; tergite 7 is missing, and tergite 8 is reduced to a bare lateral plate (Fig. 6c). Sternite 7 is somewhat grooved in the middle and lobulated, nearly bare, excluding the posterior margin, which has scarce hairs and stout bristles; the 8^th^ sternite is well-developed, bare, and membranous; it has triangular cerci with long hairs. Three highly sclerotized spermathecae are present (Figs. 6d, e, 7).

### Molecular identification

In this work, species identification of the flies based on morphological characteristics was validated through DNA barcoding to ensure accurate species identification. Amplification of the COI gene from *S. ruficornis* produced an 817 bp fragment, which was submitted to GenBank^4^ under the accession numbers OQ077960 (F) and OQ077961 (M). BLAST analysis on NCBI revealed that sequencing of the targeted COI gene showed 100% identity with the Indian *S. ruficornis* (KX230686) and a 99.66% identity with the *S. ruficornis* complete mitochondrial genome (MH937749.1). Additionally, analysis of the COI sequence as an unknown sample in the BOLD system confirmed the species identification with a 100% similarity to the same Indian *S. ruficornis*, which is consistent with the NCBI results.

A search of the NCBI and BOLD databases identified 63 and 74 published records of *S. ruficornis*, respectively. The difference of 11 records was due to direct submissions from various institutions to the BOLD database, namely: the University of Wollongong (6), the Research Collection of Graeme V. Cocks (2), National Institute for Biotechnology and Genetic Engineering (1), the Gujarat State Biotechnology Mission (1), and National Bureau of Agricultural Insect Resources, India (1).

**Fig. 8:**
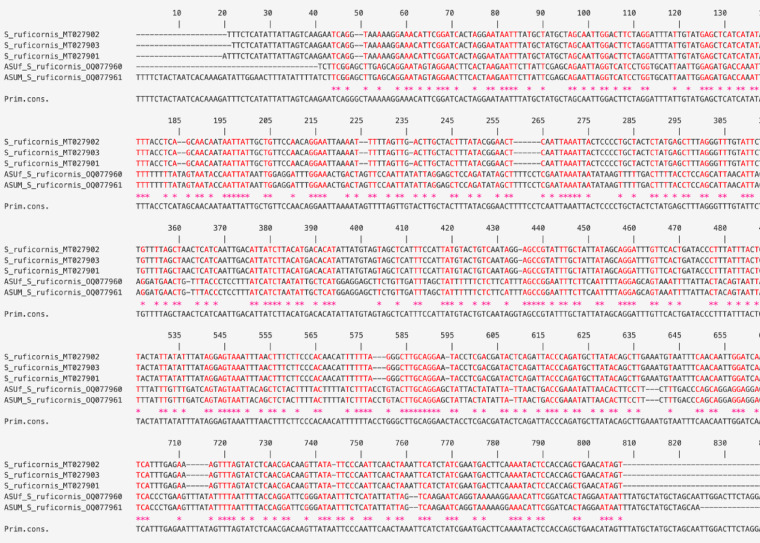
Multiple sequence alignment of *S. ruficornis* sequences from this study (OQ077960, OQ077961) and three sequences from Egypt (MT027901–MT027903) retrieved from GenBank. The alignment reveals significant genetic divergence, with a pairwise similarity of less than 39.26%. The visualization indicates conserved regions, nucleotide mismatches, and insertion/deletion gaps, illustrating the pronounced genetic disparity between the sequence sets.

**Fig. 9:**
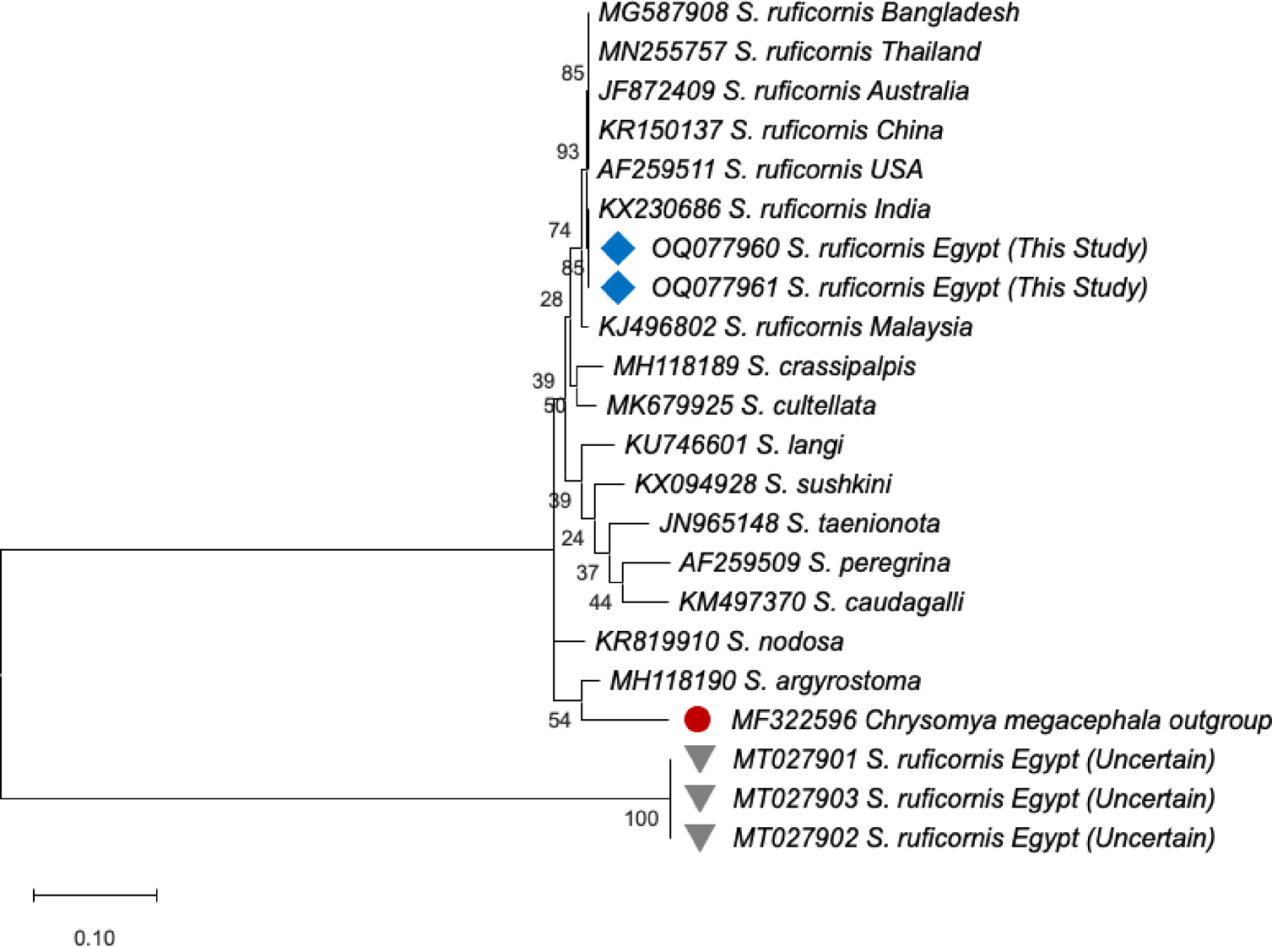
Maximum Likelihood phylogenetic tree of *Sarcophaga ruficornis* and related species based on the Kimura 2-parameter model of the COI gene sequences. The tree was rooted with *Chrysomya megacephala* (Family: Calliphoridae, MF322596). The analysis includes sequences from this study (OQ077960 and OQ077961), the three putative Egyptian sequences (MT027901-MT027903), sequences of *Sarcophaga ruficornis* from other countries, and additional congeneric species. Bootstrap values (based on 1000 replicates) are shown at the nodes.

Among the published sequences, three in GenBank are listed as originating from Egypt with accession numbers: MT027901-MT027903. However, multiple sequence alignment revealed that their similarity to our sequences did not exceed 39.26% (Fig. 8). A phylogenetic tree was constructed from the sequences from this study, the three putative Egyptian sequences, and the top hits from our BLAST search using MEGA11 (Fig. 9). The phylogenetic tree was constructed using the ML method and rooted with *Chrysomya megacephala* (Family: Calliphoridae, MF322596). This species was selected as the outgroup because Calliphoridae represents a closely related family to Sarcophagidae within the Calyptratae dipteran clade, ensuring it shares sufficient genetic similarity for meaningful character comparison while being evolutionarily distinct enough to provide a reliable root for the tree. This approach follows established phylogenetic practice for ensuring accurate topological inference within Sarcophagidae.

Based on the COI gene sequences from various species of *Sarcophaga*; the resulted phylogenetic relationship (Fig. 9) revealed a well-supported monophyletic clade containing all confirmed sequences of *Sarcophaga ruficornis*. The sequences from this study (OQ077960 and OQ077961) clustered with conspecific sequences from Bangladesh, Thailand, China, India, and other regions, supporting their identification. Notably, and as anticipated, the three putative Egyptian sequences did not share an evolutionary history with any *Sarcophaga* species and were not phylogenetically related to any of the other results.

Estimates of evolutionary divergence based on the Kimura 2-parameter model were calculated to assess the levels of intraspecific and interspecific variation (Table 1). Pairwise Genetic divergence analysis revealed several distinct clusters. The primary cluster, representing the true *Sarcophaga ruficornis*, exhibited very low intraspecific divergence (0.0% to ~ 1.0%). This group included sequences from Australia (JF872409), Bangladesh (MG587908), Thailand (MN255757), China (KR150137), India (KX230686), Malaysia (KJ496802), the USA (AF259511), and the two sequences from this study in Egypt (OQ077960, OQ077961). The Egyptian sequences from this study were genetically identical to each other and to the specimen from India (KX230686). In stark contrast, three sequences from Egypt obtained from GenBank (MT027901, MT027902, MT027903) were highly divergent, showing a genetic distance of approximately 95.0–99.0% from the true *Sarcophaga ruficornis* cluster. As expected, comparisons to other congeneric *Sarcophaga* species (e.g., *S. sushkini, S. taenionota, S. caudagalli, S. argyrostoma, S. langi, S. nodosa, S. crassipalpis, S. cultellata)* showed intermediate interspecific divergence, ranging from approximately 3.0–8.0%. All *Sarcophaga* sequences were clearly distinct from the outgroup, *Chrysomya megacephala*, which showed the highest genetic distances (approximately 10.0–16%).

**Table 1:** Estimates of evolutionary divergence between sequences based on the Kimura 2-parameter (K2P) model for an 817 bp fragment of the cytochrome c oxidase subunit I (COI) gene.

		1	2	3	4	5	6	7	8	9	10	11	12	13	14	15	16	17	18	19	20	21	22
1	AF259509_*S._peregrina*		0.01	0.01	0.01	0.01	0.01	0.09	0.09	0.08	0.01	0.01	0.01	0.01	0.01	0.01	0.01	0.01	0.01	0.01	0.01	0.01	0.02
2	JF872409_*S._ruficornis_*Australia	0.08		0.00	0.00	0.00	0.00	0.08	0.08	0.08	0.00	0.00	0.00	0.00	0.01	0.01	0.01	0.01	0.01	0.01	0.01	0.01	0.01
3	MG587908_*S._ruficornis*_Bangladesh	0.08	0.00		0.00	0.00	0.00	0.08	0.08	0.08	0.00	0.00	0.00	0.00	0.01	0.01	0.01	0.01	0.01	0.01	0.01	0.01	0.01
4	KJ496802_*S._ruficornis*_Malaysia	0.08	0.01	0.01		0.00	0.00	0.08	0.08	0.07	0.00	0.00	0.00	0.00	0.01	0.01	0.01	0.01	0.01	0.01	0.01	0.01	0.01
5	MN255757_*S._ruficornis*_Thailand	0.08	0.00	0.00	0.01		0.00	0.08	0.08	0.08	0.00	0.00	0.00	0.00	0.01	0.01	0.01	0.01	0.01	0.01	0.01	0.01	0.01
6	KR150137_*S._ruficornis*_China	0.08	0.00	0.00	0.01	0.00		0.08	0.08	0.08	0.00	0.00	0.00	0.00	0.01	0.01	0.01	0.01	0.01	0.01	0.01	0.01	0.01
7	MT027903_*S._ruficornis*_Egypt_(Uncertain)	1.00	0.97	0.97	0.95	0.97	0.97		0.00	0.00	0.08	0.08	0.08	0.08	0.08	0.08	0.08	0.09	0.08	0.08	0.08	0.08	0.09
8	MT027902*_S._ruficornis*_Egypt_(Uncertain)	1.00	0.97	0.97	0.95	0.97	0.97	0.00		0.00	0.08	0.08	0.08	0.08	0.08	0.08	0.08	0.09	0.08	0.08	0.08	0.08	0.09
9	MT027901_*S._ruficornis*_Egypt_(Uncertain)	1.02	0.99	0.98	0.97	0.99	0.99	0.00	0.00		0.08	0.08	0.08	0.08	0.08	0.08	0.08	0.08	0.08	0.08	0.08	0.08	0.08
10	KX230686_*S._ruficornis_*India	0.09	0.00	0.00	0.01	0.00	0.00	0.97	0.97	0.97		0.00	0.00	0.00	0.01	0.01	0.01	0.01	0.01	0.01	0.01	0.01	0.01
11	OQ077960_*S._ruficornis*_Egypt_(This Study)	0.09	0.00	0.00	0.01	0.00	0.00	0.97	0.97	0.97	0.00		0.00	0.00	0.01	0.01	0.01	0.01	0.01	0.01	0.01	0.01	0.01
12	OQ077961_*S._ruficornis*_Egypt_(This Study)	0.09	0.00	0.00	0.01	0.00	0.00	0.96	0.96	0.96	0.00	0.00		0.00	0.01	0.01	0.01	0.01	0.01	0.01	0.01	0.01	0.01
13	AF259511_*S._ruficornis*_USA	0.08	0.00	0.00	0.01	0.00	0.00	0.97	0.97	0.97	0.00	0.00	0.00		0.01	0.01	0.01	0.01	0.01	0.01	0.01	0.01	0.01
14	KX094928_*S._sushkini*	0.07	0.06	0.06	0.06	0.06	0.06	0.97	0.97	0.99	0.06	0.06	0.06	0.06		0.01	0.01	0.01	0.01	0.01	0.01	0.01	0.02
15	JN965148_*S._taenionota*	0.07	0.06	0.06	0.06	0.06	0.06	0.97	0.97	1.00	0.06	0.06	0.06	0.06	0.06		0.01	0.01	0.01	0.01	0.01	0.01	0.01
16	KM497370_*S._caudagalli*	0.07	0.06	0.06	0.06	0.06	0.06	0.96	0.96	0.98	0.06	0.06	0.06	0.06	0.08	0.07		0.01	0.01	0.01	0.01	0.01	0.02
17	MH118190_*S._argyrostoma*	0.09	0.06	0.06	0.06	0.06	0.06	0.99	0.99	1.00	0.06	0.06	0.06	0.06	0.08	0.07	0.08		0.01	0.01	0.01	0.01	0.01
18	KU746601_*S._langi*	0.08	0.06	0.06	0.05	0.06	0.06	0.96	0.96	0.98	0.06	0.06	0.06	0.06	0.06	0.08	0.08	0.07		0.01	0.01	0.01	0.01
19	KR819910*_S._nodosa*	0.09	0.05	0.05	0.04	0.05	0.05	0.99	0.99	1.01	0.05	0.05	0.05	0.05	0.08	0.08	0.08	0.06	0.07		0.01	0.01	0.01
20	MH118189_*S._crassipalpis*	0.09	0.04	0.04	0.04	0.04	0.04	0.98	0.98	1.00	0.04	0.04	0.04	0.04	0.07	0.07	0.08	0.07	0.06	0.06		0.01	0.02
21	MK679925_*S._cultellata*	0.08	0.03	0.03	0.03	0.03	0.03	0.99	0.99	0.98	0.03	0.03	0.03	0.03	0.06	0.06	0.08	0.06	0.06	0.05	0.04		0.01
22	MF322596_*Chrysomya_megacephala_*outgroup	0.16	0.11	0.10	0.13	0.11	0.11	1.03	1.03	1.03	0.10	0.10	0.10	0.10	0.14	0.12	0.13	0.10	0.13	0.14	0.14	0.12	

## Discussion

Typically, dipteran insects, specifically some species of the Sarcophagidae, are very important in forensic entomology^1^, with the primary use being the calculation of the minPMI. Accurate species identification is one of the crucial pieces of information required to calculate the minPMI correctly. Thus, the present work focuses on the identification of *Sarcophaga ruficornis*, a fly of forensic interest, through an integrative taxonomic approach that combines both morphological and molecular methods.

The genus *Sarcophaga* Meigen (1826) is highly biodiverse, and its classification has undergone frequent changes since its initial description. Kurahashi and Samerjai^29^ reclassified *S. ruficornis* as *Liopygia ruficornis* in their comprehensive taxonomic work on the fauna of Sarcophagidae in Thailand. In a notable effort to simplify the classification of the diverse *Sarcophaga* genus, they elevated *Liopygia* Enderlein, 1928 to the generic level, distinguishing it from other genera of the tribe Sarcophagini by its gena, which is almost entirely covered in yellowish-white hairs. However, aside from some highly divergent opinions on ranking, modern researchers appear to largely align with the World Catalogue’s classification^30^ in which the genus *Sarcophaga* serves as a large, inclusive genus encompassing various morphologically distinct but phylogenetically related groups divided into subgenera, and the classification within the genus depends mainly on the detailed structure of the male genitalia. Consequently, *Liopygia* was considered valid as a subgenus under *Sarcophaga* according to the Biosystematics database of Order Diptera^31^. In Egypt, the genus *Sarcophaga* is represented by 27 species within nine subgenera, and a taxonomic key has been provided to distinguish between 21 of them^32^. Confirmation of the presence of *S. ruficornis* in the current study brings the total number of species to 28.

In the current work, the successful colonization of the species in the laboratory facilitated the study of the molecular and morphological characteristics of both males and females using appropriately preserved fresh specimens.

Indeed, the identification of male Sarcophagidae is straightforward, and is predominantly based on the examination of their genitalia^8,33^, although it can still be challenging! The phallus, which includes the aedeagus and associated structures, is often the most reliable morphological feature for distinguishing between closely related species within this family. The examination of male genitalia requires careful analysis of subtle variations in the shape, size, and configuration of these structures^30^. However, it is often difficult to observe these characteristics due to the improper preservation of specimens gathered during forensic investigations^10^.

The current results revealed that males of *S. ruficornis* can be differentiated from those of closely related species belonging to the subgenus *Liopygia* by the following characters: antennal flagellum reddish yellow, parafacial black with golden pollen, gena black colored with silver pollen and yellow hairs, 12 frontal bristles, R_1_ bare, first genital segment reddish-brown with long marginal bristles, second with black hairs with no marginal bristles, cerci with acute pointed end, densely covered with elongated hair; surstylus slightly ovate with apical hairs and ventralia bulbous.

Although sarcophagid female specimens are often more abundant on carcasses, the morphological similarities among species often hinder precise identification, as the female genitalia do not typically exhibit distinctive taxonomic characteristics^15,23,34^. This often necessitates identifying females based on their association with co-occurring males, a method that is not always feasible or reliable at crime scenes. Few studies have been conducted on the female genitalia of sarcophagids; Sukontason^35^ on *Sarcophaga dux*, Szpila et al.^36^ on *S. albiceps*, *S. carnaria, S. argyrostoma*, *S. caerulescens*, *S. similis,* and *S. melanura,* and Vairo et al.^34^ on nine species within four genera, including *Sarcophaga africa*. In this work, the genitalia of female *S.ruficornis* were dissected and illustrated for the first time in Egypt.

The current results revealed that tergite 6 is undivided, a characteristic that it resembles *Oxysarcodexia riograndensis*, *Peckia lambens*, and *Microcerella halli*^34^, while the 7th tergite is commonly absent, and the 8th tergite is typically reduced to bare lateral plates as is common in Sarcophagidae^37^. Additionally, the epiproct is not apparent, the hypoproct is not sclerotized, sternite 7 is slightly grooved medially and lobulated, and sternite 8 is well-differentiated and membranous. These characters are similar to those of *Sarcophaga mailansis* reported by Kumar et al.^38^. The fusion or absence of sternites 6, 7, and 8 provides significant information for differentiating sarcophagid females, although the shape of sternites can be a useful characteristic in general. The shape of the spermathecae is another diagnostic factor for distinguishing genera and subgenera. In the present work, *S. ruficornis* was found to have three highly sclerotized, elongated spermathecae, while they are rounded in *P. lambens* and *Peckia florencioi*^34^.

Thus, this meticulous dissection process is essential for accurate sarcophagid species identification based on morphology, but it demands a high level of expertise and experience, limiting its accessibility to non-specialist forensic practitioners^36,39^. Furthermore, morphological identification can be challenging, particularly due to the absence of accessible local taxonomic keys and the difficulty in applying them, especially for juvenile stages. Additionally, damaged specimens may render certain taxonomic keys unusable for accurate identification. Conversely, molecular methods offer a more reliable technique for the identification of forensically significant insects irrespective of their condition or life stage. Specific molecular markers are recognized as valuable tools for molecular identification because studies have shown that interspecific variation often exceeds intraspecific variation for certain genes, providing a reliable basis for species identification^7^.

The principle behind using molecular markers for DNA barcoding is to detect genetic differences between species. Mitochondrial DNA (mtDNA) is particularly preferred for barcoding due to its high mutation rate within species and relatively low variation between species. Among these markers, the COI gene is one of the most widely used and effective tools for species identification, as it is highly conserved within species while allowing differentiation between species^7^.

The mitochondrial COI gene has been widely recognized as a primary candidate for identifying forensically important insects^8,16,23^. However, despite over 20 years of using molecular data for the identification of forensic insects, inconsistencies in gene selection and phylogenetic methodologies across studies continue to hinder the efficiency of this approach^40^. Before employing this gene in actual forensic entomology cases, it is essential to assess the suitability of specific COI gene markers, such as the 272-bp and 1173-bp fragments, for species identification within specific geographic regions^41,42^.

To our knowledge, the barcode sequence of the mitochondrial COI gene from *S. ruficornis* in this study, which yielded an amplified fragment of 817 bp, is the first definitive molecular data for the forensically important *S. ruficornis* from Egypt. The sequences from this study group were grouped within the same clade as *S. ruficornis* from various countries, forming a monophyletic group that confirms their close evolutionary relationship. One unexpected result was the relatively low similarity (~ 39%) between our samples and three Egyptian *Sarcophaga* sequences in GenBank. The putative Egyptian sequences, however, formed a distinct cluster, showing no phylogenetic affiliation with any *Sarcophaga* species. This discrepancy may be due to errors in species identification or mislabeling during database submission. Such issues are known to occur in public repositories and represent a limitation when relying on secondary data. While our findings confirm the usefulness of the COI marker, they also highlight the need for validated reference sequences generated from carefully identified specimens to improve the accuracy of future molecular identification studies.

Furthermore, the results clearly define a robust molecular cluster for *S. ruficornis*, which exhibits minimal genetic divergence across a wide geographic range that includes Australia, Asia, North America, and now, as confirmed by this study, Egypt. The two Egyptian sequences generated here are unequivocally part of this cluster, demonstrating the presence of this species in the region. The most significant finding concerns the three previously published Egyptian sequences (MT027901-MT027903), which are genetically divergent to a degree (~ 95–99%) that precludes their classification as *Sarcophaga ruficornis* or even a close congener. This extreme divergence, exceedingly even the distance to the outgroup *Chrysomya megacephala*, strongly indicates a profound misidentification of the source specimen(s). The data from this study therefore serve to correct the taxonomic record for Egypt. The observed pattern of divergence between *Sarcophaga ruficornis* and other species within the genus (3–8%) is consistent with expected levels for congeneric species, while the greater distance to the outgroup *Chrysomya megacephala* (10–16%) validates the use of the COI gene for distinguishing among genera and beyond.

## Conclusions

The present study successfully identified *S. ruficornis* using both morphological analysis and DNA barcoding, confirming its presence and documenting its occurrence in the Egyptian fauna. Overall, the findings of this investigation enrich the molecular data on Sarcophagidae in Egypt and thereby contribute to and expanding the existing global knowledge of sarcophagid flies and facilitating further research into their use in crime scene investigation.

## Data Availability

The datasets generated and/or analysed during the current study are available in the NCBI GenBank repository under accession numbers OQ077960 and OQ077961. These data can be accessed at https://www.ncbi.nlm.nih.gov/nuccore/OQ077960 and https://www.ncbi.nlm.nih.gov/nuccore/OQ077961.

## Abbreviations

BLAST: Basic local alignment search tool
BOLD: Barcode of life data
Bp: Base pair
COI: The mitochondrial cytochrome oxidase subunit one gene
F: Female
KOH: Potassium hydroxide
K2P: Kimura 2-parameter
LD: Light/Dark
M: Male
MEGA: Molecular evolutionary genetics analysis
minPMI: Minimum postmortem interval
ML: Maximum likelihood
NCBI: National center for biotechnology information
*P. lambens*: *Peckia lambens*
R_1_: Radius_1_
*S.*: *Sarcophaga*
